# Molecular Bases of VEGFR-2-Mediated Physiological Function and Pathological Role

**DOI:** 10.3389/fcell.2020.599281

**Published:** 2020-11-16

**Authors:** Xinrong Wang, Alfredo Maria Bove, Giuseppe Simone, Binyun Ma

**Affiliations:** ^1^College of Animal Science and Technology, Gansu Agricultural University, Lanzhou, China; ^2^Regina Elena National Cancer Institute, Rome, Italy; ^3^Department of Medicine/Hematology, Keck School of Medicine of the University of Southern California, Los Angeles, CA, United States

**Keywords:** VEGF, VEGFR-2, structure, function and role, vasculogenesis, angiogenesis

## Abstract

The vascular endothelial growth factors (VEGFs) and their receptors (VEGFRs) play crucial roles in vasculogenesis and angiogenesis. Angiogenesis is an important mechanism in many physiological and pathological processes, and is involved in endothelial cell proliferation, migration, and survival, then leads to further tubulogenesis, and finally promotes formation of vessels. This series of signaling cascade pathways are precisely mediated by VEGF/VEGFR-2 system. The VEGF binding to the IgD2 and IgD3 of VEGFR-2 induces the dimerization of the receptor, subsequently the activation and trans-autophosphorylation of the tyrosine kinase, and then the initiation of the intracellular signaling cascades. Finally the VEGF-activated VEGFR-2 stimulates and mediates variety of signaling transduction, biological responses, and pathological processes in angiogenesis. Several crucial phosphorylated sites Tyr801, Try951, Try1175, and Try1214 in the VEGFR-2 intracellular domains mediate several key signaling processes including PLCγ-PKC, TSAd-Src-PI3K-Akt, SHB-FAK-paxillin, SHB-PI3K-Akt, and NCK-p38-MAPKAPK2/3 pathways. Based on the molecular structure and signaling pathways of VEGFR-2, the strategy of the VEGFR-2-targeted therapy should be considered to employ in the treatment of the VEGF/VEGFR-2-associated diseases by blocking the VEGF/VEGFR-2 signaling pathway, inhibiting VEGF and VEGFR-2 gene expression, blocking the binding of VEGF and VEGFR-2, and preventing the proliferation, migration, and survival of vascular endothelial cells expressing VEGFR-2.

## Introduction

In organism, various physiological and pathological processes are involved in vasculogenesis, angiogenesis, and formation and maintenance of new blood vessel structures, including embryonic development (Vallon et al., 2014), tissue growth and wound healing (Ivkovic et al., 2003), tumorigenesis (Yehya et al., 2018), rheumatoid arthritis (Marrelli et al., 2011), diabetic retinopathy (Crawford et al., 2009; Cheng and Ma, 2015), axon growth (Klagsbrun and Eichmann, 2005), cancer (Hanahan and Folkman, 1996; Rajabi and Mousa, 2017; Li et al., 2020), and inflammation (Alkim et al., 2015), which are stimulated by a variety of factors, including basic fibroblast growth factor (bFGF) (Iwasaki et al., 2004), vascular endothelial growth factor (VEGF) (Hoeben et al., 2004; Nilsson and Heymach, 2006), platelet-derived growth Factor (PDGF) (Lindahl et al., 1999), ephrin-Eph receptors (Zhang and Hughes, 2006), angiopoietin-1 (Yin et al., 2020), hepatocyte growth factor (HGF) (Kaga et al., 2012), transforming growth factor-β (TGF-β) (Ferrari et al., 2009), and interleukin 6 (IL-6) (Gopinathan et al., 2015), etc. VEGFs and their receptors (VEGFRs) are currently the most important and specific factors to stimulate endothelial cell proliferation, regulate both the development of blood vessels from precursor cells during early embryogenesis and the formation of blood vessels from pre-existing vessels at a later stage, and increase vascular permeability and chemotaxis of vascular endothelial cells (Ferrara and Kerbel, 2005; Benedito et al., 2012; Chen et al., 2013). The VEGF and its receptor VEGFR have been reported to play crucial roles not only in physiological but in most pathological angiogenesis.

Vascular endothelial growth factors are important signaling molecules involved in both vasculogenesis and angiogenesis that are the two distinct processes by which new vascular network are formed during embryonic development (Drake et al., 2000). The vasculogenesis is a fundamental process of blood vessel system formation in the embryo, occurring by a *De novo* synthesis and differentiation of endothelial precursor cells into endothelial cells, and it is the first stage of the formation of the vascular network. The angiogenesis is a vital physiological process of growth of new capillaries through which the pre-existing vasculatures formed in the earlier stage of the vasculogenesis continue to grow, sprout, split, and grow (Drake et al., 2000). VEGF is crucial to ensure normal vascular morphogenesis, especial to increase the number of capillaries in angiogenesis. The embryos lacking a single VEGF allele exhibit abnormal vascular development and lethality (Carmeliet et al., 1996; Ferrara et al., 1996). The VEGF and its receptor VEGFR have been shown to play important roles in many angiogenic processes not only in normal physiological conditions but in most pathological conditions, such as embryonic development, axon growth, cancer, and inflammation (Hanahan and Folkman, 1996; Risau, 1997; Bellon et al., 2010). VEGF is a sub-family of the cystine-knot growth factor PDGF supergene family. All members of the VEGF family can stimulate cellular responses by binding to their tyrosine kinase receptors (VEGFRs) on the cell surface.

Currently, the human VEGF/VEGFR system is composed of VEGF-A (also VEGF), VEGF-B, VEGF-C, VEGF-D, and PGF (placental growth factor), three main VEGF receptors VEGFR-1 (Flt-1), VEGFR-2 (KDR), VEGFR-3 (Flt-4), and two non-protein kinase co-receptors neuropilin-1 and neuropilin-2 (NRP-1 and -2) (Rahimi, 2006; Shibuya, 2011). The VEGFR-1 and VEGFR-2 regulate angiogenesis and vascular permeability, and the VEGFR-3 mainly regulates lymphangiogenesis (Alitalo and Carmeliet, 2002). Among them, VEGFR-2 is mainly distributed in vascular endothelial cells and acts as major signal transducer for angiogenesis by PLCγ-PKC-MAPK, PLCγ-PKC-eNOS-NO, TSAd-Src-PI3K-Akt, SHB-FAK-paxillin, SHB-PI3K-Akt, and NCK-p38-MAPKAPK2/3 pathways (Wong and Jin, 2005; Shibuya, 2011). The VEGF/VEGFR system is an important target for anti-angiogenic therapy in cancer and for pro-angiogenic therapy in neuronal degeneration and ischemic diseases (Yang et al., 2018). Here, the molecular structures, physiological functions, and pathological roles of VEGFR-2 and its regulation mechanisms of signal transduction have been analyzed and reviewed.

## VEGFR-2 Structural Characteristics and Function

Vascular endothelial growth factors are main regulators in vasculogenesis and angiogenesis and play their roles by binding to VEGFRs on cell surface and activating subsequently the signaling pathways of angiogenesis. The VEGFR-2 is the major receptor of VEGF, expresses in vascular endothelial cells, and plays a major role in angiogenesis (Bellon et al., 2010; Leppanen et al., 2010).

### VEGFR-2 Molecular Characteristics

**FIGURE 1:**
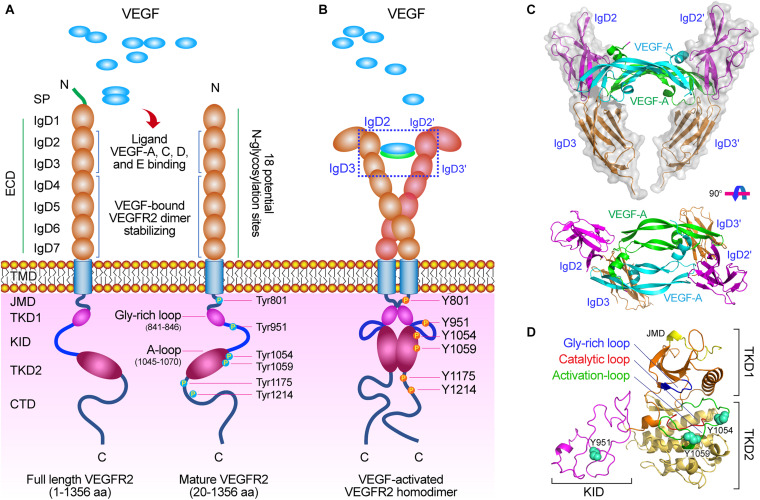
The molecular structure of VEGF/VEGFR-2. **(A)** Diagram of the VEGFR-2 structure. VEGFR-2 is composed of a signal peptide, a extracellular domain (ECD) including seven Ig-like subdomains (IgD1∼7), a TMD, a JMD, a catalytic tyrosine kinas domain (TKD) including ATP binding domain (TKD1), kinase insert domain (KID) and phosphotransferase domain (TKD2), and a flexible C-terminal domain (CTD), and many functional sites. **(B)** VEGF-activated VEGFR-2 homodimer. After VEGFs binding to VEGFR-2, the crucial tyrosine residues on the TKD have been phosphorylated and are involved in mediating downstream signaling pathways. **(C)** Molecular structure of VEGF-A binding to IgD2 and IgD3 of VEGFR-2 [PDB ID: 3V2A (Brozzo et al., 2012)]. **(D)** Molecular structure of TKD of VEGFR-2 including TKD1 (N-lobe), KID, and TKD2 (C-lobe) [PDB ID: 4ASD (McTigue et al., 2012)]. There are three important motifs: glycine-rich loop (blue, 841–846 aa), catalytic loop (red, 1026–1033 aa), and activation loop (green, 1045–1075 aa), and three crucial phosphorylation sites (spheres) on the TKD: Tyr951 on the KID, and Tyr1054 and Try1059 on the TKD2.

Human VEGFR-2, a kinase insert domain containing receptor (KDR) gene, is located at chromosome locus 4q11-12 and encodes 1356 amino acids of the full-length receptor (Park et al., 2018). Mature VEGFR-2 is a transmembrane glycoprotein with a molecular weight of 230 kD (Figure 1; Takahashi and Shibuya, 1997). The other two forms of VEGFR-2 are the non-glycosylated form with a molecular weight of 150 kD and the intermediate form with a molecular weight of 200 kD (Shen et al., 1998). Only the mature glycosylated form of VEGFR-2 (KDR) can achieve intracellular signal transduction (Shibuya, 2013). Mouse VEGFR-2 is also called fetal liver kinase 1 (Flk-1), which is composed of 1367 amino acids and has 83% homology with human KDR, and has three forms, molecular weight of 180, 200, and 220 kD, respectively.

VEGFR-2 is mainly distributed in vascular endothelial cells, lymphatic endothelial cells, and embryonic precursor cells, and can bind to VEGF-A, VEGF-C, and VEGF-D. By binding and activating VEGFR-2, VEGF mediates endothelial cell proliferation, invasion and migration, and survival, and increases vascular permeability and neovascularization (Holmes et al., 2007).

### VEGFR-2 Molecular Structure

The full-length VEGFR-2 is composed of 1356 amino acids including a signal peptide (1∼19 aa) and a mature protein (20∼1356 aa). As a mature transmembrane protein, the VEGFR-2 is divided into extracellular domain (ECD, 20∼764 aa), transmembrane domain (TMD, 765∼789 aa), juxtamembrane domain (JMD, 790∼833 aa), catalytic tyrosine kinas domain (TKD, 834∼1162 aa) including ATP binding domain (TKD1, 834∼930 aa), kinase insert domain (KID, 931∼998 aa) and phosphotransferase domain (TKD2, 999∼1162 aa), and a flexible C-terminal domain (CTD, 1163∼1356 aa) (Table 1 and Figure 1).

**TABLE 1:** The structures and functions of the VEGFR-2 domains.

**Domains**	**Structure characteristic**	**Functions and roles**
ECD	20∼764 aa	Plays a central role in VEGF binding, trafficking, stabilizing, and pro-angiogenic signaling in physiological and pathological condition by the IgD2 and IgD3, and the *N*-glycosylation sites.
	The extracellular domain including 7 Ig-like subdomains (IgD1∼7) connected by the linkers, and 18 potential *N*-glycosylation sites	
TMD	765∼789 aa	Regulates VEGFR-2 kinase activity, specific orientation of the intracellular kinase domains in active VEGFR-2 dimers, and dimerization of the receptor monomers with specific orientations.
	A single transmembrane α-helical	
JMD	790∼833 aa	Plays a crucial role for the autophosphorylation rate of VEGFR-2. Unphosphorylated JMD autoinhibits kinase activity by the site Y801 interacting with the activation loop in the kinase domain.
	A flexible intracellular regulatory region including the first phosphorylation site Y801 of VEGFR-2	
TKD	834∼1162 aa	Contains the catalytic site of VEGFR-2 and is involved in VEGFR-2 activation and signaling by its multiple phosphorylation sites and VEGFR-2-mediate cellular signaling, and regulation of endothelial cell survival, proliferation, cell migration, and the vascular tube formation.
	The catalytic tyrosine kinas domain contains three subdomains: • TKD1 (834∼930aa), an ATP binding domain including a hydrophobic pocket with a glycine-rich motif • KID (931∼998aa), a kinase insert domain including a phosphorylation site Y951 • TKD2 (999∼1162aa), a phosphotransferase domain including two sites Y1054 and Y1059, a catalytic loop and an activation loop	
CTD	1163∼1356 aa	Is critical for VEGFR-2 activation and signaling and is involved in VEGFR-2-mediate cellular signaling and endothelial cell survival, proliferation, cell migration, and permeability of vascular endothelial cells.
	The carboxyl terminus domain including two important autophosphorylation sites Y1175 and Y1214	

The ECD is composed of 7 immunoglobulin-like subdomains, IgD1 (46∼110 aa), IgD2 (141∼207 aa), IgD3 (224∼320 aa), IgD4 (328∼414 aa), IgD5 (421∼548 aa), IgD6 (551∼660 aa), and IgD7 (667∼753 aa) (Figure 1A; Fuh et al., 1998). Investigation have showed that the IgD1 is involved in regulating the binding of receptors to ligands; while the IgD2 and IgD3 are required for tight binding to the dimeric VEGF and VEGF-induced VEGFR-2 dimerization and activation; the IgD2 and IgD4 can affect the binding rate of the ligand; the IgD5 and IgD6 may be involved in affect the receptor molecules unbinding from the ligands; and the IgD7 plays a crucial role in receptor dimerization and regulation (Figures 1B,C; Shinkai et al., 1998; Di Stasi et al., 2019).

The TKD is the most conserved region among VEGFRs (Park et al., 2018). This protein kinase core of the VEGFR-2 has a two-lobed spatial structure that forms the active center between the both lobes. At the N-terminus of the intracellular tyrosine kinas domain, there is a hydrophobic pocket containing a glycine-rich (GXGXXG, 841∼846 aa) ATP phosphate binding loop in the β-sheet structures. At the TKD C-terminal, there are several α-helical structures, including a catalytic loop (HRDLAARN, 1026∼1033 aa), and activation loop (A-loop, 1045∼1075 aa), which play important roles for VEGFR-2 catalytic properties (Tables 1, 2 and Figures 1A,D).

**TABLE 2:** Functional sites and motifs in VEGFR2.

**Sites and motifs**	**Location**	**Function**
**N-linked glycosylation sites:**		
Asn46, 66, 96, 143, 158, 245, 318, 374, 395, 511, 523, 580, 613, 619, 631, 675, 704, and 721	ECD	These sites play a central role in RTK ligand binding, trafficking, stabilizing, and pro-angiogenic signaling in physiological and pathological contexts.
**Phosphorylation sites:**		
Tyr801	JMD	Y801 is crucial for the autophosphorylation rate of VEGFR-2, and phosphorylated Y801 disrupts the interaction of JMD with the A-loop, promotes reorientation of the activation loop, and induces an enzymatically active conformation.
Tyr951	KID	Y951 is phosphorylated on VEGFA stimulation, and phosphorylated Y951 at the TKD of VEGFR-2 can bind TSAd, activate formation of TSAd and Src complex, subsequently regulate cell migration and mediate cell survival and permeability.
Tyr1054	TKD2	Y1054 and Y1059 sites are located in the TKD2 A-loop and play crucial role in kinase activity, and phosphorylated Y1054 and Y1059 increase the VEGFR-2 kinase activity.
Tyr1059		
Tyr1175	CTD	Y1175 and Y1214 are critical for VEGFR-2 activation and signaling and are involved in VEGFR-2-mediate cellular signaling, and phosphorylated Y1175 mediates the binding of VEGFR-2 with PLCγ, PI3K, and adapter proteins SHB and SCK and phosphorylated Y1214 mediates the binding of VEGFR-2 with adapter protein NCK, and the both of pY1175 and pY1214 finally directly activate VEGFR-2 to promote the proliferation, migration, and permeability of vascular endothelial cells.
Tyr1214		
**Others phosphorylation sites:**		
Ser982, Ser984, and Tyr996	KID	They are potential phosphorylation sites and may be involved in VEGFR-2 activation and signaling.
Ser1231, Ser1235, and Thr1238	CTD	
Tyr1305, Tyr1309, and Tyr1319	CTD	
**Functional motifs:**		
Glycine-rich (G^841^XGXXG) (841∼846 aa)	TKD1	The glycine-rich forms a functionally important ATP phosphate binding loop, which is a flexible segment whose position differs among kinase structures in various activated and liganded states.
Catalytic loop (H^1026^RDLAARN) (1026∼1033 aa)	TKD2	The catalytic loop contains an invariant aspartic acid residue (D1028) that is essential for catalysis of the phosphotransferase reaction. The catalytic loop is highly conserved among protein tyrosine kinases.
Activation loop (A-loop) (1045∼1075 aa)	TKD2	The activation loop is a large flexible loop in protein kinases and contains two tyrosines Y1054 and Y1059, whose conformation is postulated to regulate kinase activity.

In the human VEGFR-2, there are 18 N-linked glycosylation sites, 15 phosphorylation sites, and many ATP binding sites and substrate binding sites, which play important roles in post-translational modifications of VEGFR-2, protein folding, protein activation, and cellular attachment, and can further modulate the function of VEGFR-2 (Croci et al., 2014; Chandler et al., 2019; Chung et al., 2019).

### Mechanism of VEGFR-2 Activation

**FIGURE 2:**
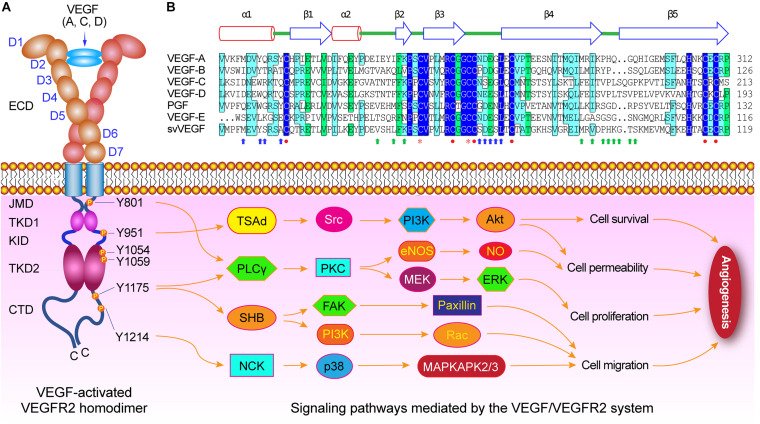
VEGF/VEGFR-2 mediated signaling pathways during angiogenesis. **(A)** Diagram of VEGF-activated VEGFR-2 homodimer. Several crucial tyrosine residues have been phosphorylated after VEGFs binding to VEGFR-2. The Try801 on JMD is involved in cell permeability and proliferation by mediating the PLCγ-PKC, then eNOS-NO or MEK-ERK, respectively. The Try951 on KID mediates the cell survival and permeability via the TSAd-Src-PI3K-Akt pathway. The Try1054 and Try1059 on TKD2 can increase VEGFR-2 kinase activity. The Try1175 is involved in cell permeability, proliferation, and migration by regulating PLCγ-PKC, SHB-FAK-paxillin, and SHB-PI3K-Rac pathways. The Try1214 mediates cell migration through NCK-p38-MAPKAPK2/3 pathway. **(B)** Structure-based sequence alignment of PDGF-derived regions of the VEGF family members, includes human VEGF-A (NP_003367), human VEGF-B (NP_003368), human VEGF-C (NP_005420), human VEGF-D (NP_004460), human PGF (placental growth factor) (NP_002623), *Orf virus* orfVEGF (VEGF-E) (ABA00650), and *Snake venom* svVEGF (BAD38844), which are composed of two α-helix and five β-sheets. There are functional sites, including receptor binding site 1 (blue arrow) and site 2 (green arrows), cysteine knot motifs (red closed circles) that form three disulfide bridges including Cys263-Cys308, Cys267-Cys310, and Cys232-Cys274 in VEGF-A, and dimerization interface sites (orange stars).

VEGFR-2 is activated by VEGF-A, -C, and -D binding to its Ig-like domains 2 and 3 (Figure 1C). Similar to other receptor tyrosine kinases (RTKs), cellular signaling mediated by VEGFR-2 is stimulated and initiated upon binding of its ligand dimer to the extracellular receptor Ig-like domains 2 and 3 (Ruch et al., 2007). This ligand-receptor interaction causes VEGFR-2 homo- and hetero-dimerization followed by phosphorylation of specific tyrosine residues located in the intracellular region including juxtamembrane domain, tyrosine kinas domain, and the carboxy-terminal domain. Subsequently, a variety of signaling molecules are recruited to VEGFR-2 dimers that activate downstream signaling pathways, and ultimately affect the physiological characteristics of endothelial cells and the entire vascular environment (Figure 2A; Fuh et al., 1998; Ruch et al., 2007; Ma et al., 2011).

### The ECD Is Required for VEGFR-2 Dimerization

The extracellular domain (ECD) of VEGFR-2 consists of several Ig-like subdomains, the linkers connecting these subdomains, and multiple N-linked glycosylation sites, which play important roles in formation of VEGF/VEGFR-2 system, in receptor dimerization after with ligand binding, and in maintain of the monomeric VEGFR-2 in the absence of ligand (Table 1). Each VEGF monomer is composed of two α-helix and five β-sheets that forms a central antiparallel beta sheet. In VEGF-A, a canonical cysteine knot formed by three intramolecular disulfide bridges Cys263-Cys308, Cys267-Cys310, and Cys232-Cys274, and two receptor binding sites and dimerization interface sites are critical for VEGF binding to VEGFR-2 and subsequently VEGFR-2 dimerization (Figure 2B).

In the ECD of VEGFRs, the ability to bind to its ligand is best studied at the biochemical and the structural level. For VEGFR-2, the ligands binding to VEGFR-2 required two Ig-like subdomains 2 and 3 (IgD2 and IgD3), and the stabilization of VEGF-bound VEGFR-2 dimers and VEGF-mediated VEGFR activity required Ig-like subdomains 4∼7 (IgD4∼7) (Li et al., 2000; Yang et al., 2010). As a *N-*glycosylated RTK, VEGFR-2 has 18 potential *N*-glycosylation sites in the seven Ig-like subdomains, which play a central role in RTK ligand binding, trafficking, stabilizing, and pro-angiogenic signaling in physiological and pathological contexts, including cancer (Table 2 and Figure 1; Croci et al., 2014; Chandler et al., 2017, 2019).

### The TMD and JMD Is Crucial for Regulating VEGFR-2 Activity

The transmembrane domain (TMD) and juxtamembrane domain (JMD) of VEGFR-2 have been shown to play pivotal roles in regulating VEGFR-2 kinase activity (Solowiej et al., 2009; Koch and Claesson-Welsh, 2012; Table 1).

Investigations on the role of the TMD in VEGFR-2 signaling showed that the activation of VEGFR-2 may depend on specific orientation of the receptor monomers in an active dimer resulting from VEGF-induced ECD rearrangement, and the TMD is involved in dimerization of the receptor monomers with specific orientations (Moriki et al., 2001; Bennasroune et al., 2004; Holmes et al., 2007; Manni et al., 2014b). The JMD of VEGFR-2 has the first phosphorylation site Y801, which is crucial for the autophosphorylation rate of VEGFR-2 (Solowiej et al., 2009). The unphosphorylated JMD autoinhibits kinase activity by interacting with the activation loop (A-loop) in the kinase domain 2 (TKD2). Therefore, the phosphorylated JMD at specific tyrosine residue Y801 may disrupt this interaction with the A-loop, promote reorientation of the activation loop, and induce an enzymatically active conformation (Table 2 and Figures 1A,B; Wybenga-Groot et al., 2001; Walter et al., 2007; Solowiej et al., 2009).

### The TKD Mediates VEGFR-2 Cellular Signaling

The activation of VEGFR-2 upon its VEGFs-mediated dimerization allows the TKD transphosphorylation, and then mediates cellular signaling, and regulates endothelial cell survival, proliferation, cell migration, and the vascular tube formation (Koch et al., 2011; Table 1). The VEGFR-2 TKD contains three subdomains, TKD1, an ATP binding domain, KID, the kinase insert domain, and TKD2, a phosphotransferase domain. The TKD is involved in VEGFR-2 activation and signaling by its multiple phosphorylation sites (Figures 1B,D).

In VEGFR-2 TKD, there are several major phosphorylation sites involved in cellular signaling mediated by TKD, including Tyr951 (Y951) in the KID and Try1054 (Y1054) and Try1059 (Y1059) in the TKD2, which is phosphorylated for the kinase activation (Takahashi et al., 2001; Matsumoto et al., 2005; Table 2). The Y951 site is phosphorylated on VEGFA stimulation, and the phosphorylated Y951 is indispensable for downstream signaling by the activated kinase (Matsumoto et al., 2005). The Y1054 and Y1059 sites are located in the TKD2 A-loop and play crucial role in kinase activity, whose autophosphorylation resulted by autophosphorylation at Y801 site increases the VEGFR-2 kinase activity (Figures 1B,D; Kendall et al., 1999; Solowiej et al., 2009).

### The CTD Is Critical for VEGFR-2 Activation and Signaling

The carboxyl terminus domain (CTD) is critical for VEGFR-2 activation and signaling. In VEGFR-2 CTD, there are two important autophosphorylation sites Try1175 (Y1175) and Try1214 (Y1214) (Table 1 and Figures 1B,D). After VEGF-A binding to VEGFR-2, activation of VEGFR-2 phosphorylates the Y1175 and Y1214 (Sase et al., 2009; Table 2). Then, the VEGF-activated VEGFR-2 bind to several signaling molecules such as PLCγ (Takahashi et al., 2001), PI3K (Kim et al., 2019), and adapter proteins SHB and SCK (Warner et al., 2000; Holmqvist et al., 2004) by the phosphorylated Try1175 site (pY1175), and adapter protein NCK (Lamalice et al., 2006) by the phosphorylated Try1214 site (pY1214), which directly activate VEGFR-2 to promote the proliferation, migration, and permeability of vascular endothelial cells (Figure 2A; Koch and Claesson-Welsh, 2012; Manni et al., 2014a).

## VEGFR-2 Mediated Cellular Signaling

The VEGF/VEGFR-2 signaling is essential for the development and maintenance of the organ-specific vascular systems and physiological function of many tissues and plays important roles in the pathogenesis of diseases such as cardiovascular disease and cancer. The VEGFR-2 has been proved to mediate various VEGF-stimulated cellular signal transduction including endothelial cell survival, proliferation, migration, and to enhance vascular permeability (Holmes et al., 2007; Siveen et al., 2017).

### VEGFR-2 Is Involved in Regulating the Survival of Endothelial Cells

The VEGFR-2 plays crucial roles in vascular endothelial cell survival and blood vessel formation *in vivo* (Sase et al., 2009). VEGFR-2 regulates endothelial cell survival mainly by the activation of TSAd-Src-PI3K-PKB/AKT signaling pathway (Figure 2). Depending on VEGFR-2 and subsequent activated PI3K, VEGF-A regulates the survival of human umbilical vein endothelial cells. The PI3K can catalyze creation of PIP3 from PIP2, and then phosphorylate and activate protein kinase B (PKB) and Akt pathway (PKB/Akt pathway) (Downward, 2004). The Akt directly phosphorylates two types of apoptosis proteins: Bcl-2 associated death promoter (BAD) and caspase 9, and then inhibits their apoptotic activity to ensure cell survival (Lee et al., 2014).

### VEGFR-2 Is Required for Mediating the Proliferation of Endothelial Cells

VEGFR-2 plays a critical role in endothelial cell proliferation during angiogenesis. Through the VEGFR-2 on the cell membrane, the VEGF-A, a mitogen of many endothelial cells, can transmit extracellular signals to the cytoplasm and activate a series of downstream signaling pathways, and regulate the proliferation of endothelial cells. VEGFR-2 is mainly through the PLCγ-PKC-Raf-MEK-MAPK signaling pathway, and transmits the VEGF signal to the nucleus to activate DNA synthesis and promote the proliferation of endothelial cells (Takahashi et al., 1999; Figure 2). The VEGFR-2 can bind and activate phospholipase C-γ (PLCγ) by phosphorylation of the C-terminal Y1175 of VEGFR-2, and then phosphorylate the PLCγ and enhance its catalytic activity (Takahashi et al., 2001; Sase et al., 2009). The activated PLCγ hydrolyzes phosphatidylinositol (4, 5)-bisphosphate (PIP2) and then produces diacylglycerol (DAG) and inositol 1, 4, 5-trisphosphate (IP3), in which IP3 can increase the intracellular Ca^2+^ concentration, and DAG is a physiological activator of PKC. The ERK in the PKC-Raf-MEK-ERK signaling cascade is activated by the VEGF-activated VEGFR-2, enters the nucleus, and then binds to transcription factors that induce gene expression in response to extracellular stimuli. Finally, VEGFR-2 is involved in the endothelial cell proliferation (Holmes et al., 2007; Rask-Madsen and King, 2008).

### VEGFR-2 Is Closely Related to the Migration of Endothelial Cells

The migration of endothelial cells is crucial for angiogenesis. A variety of signal pathways mediated by VEGFR-2 are related to the migration of endothelial cells. VEGFR-2 has been proved to regulate cell migration by activating SHB, NCK, and PI3K mediated pathway via the both phosphorylation sites Y1175 and Y1214 on VEGFR-2 CTD (Laramee et al., 2007; Graupera et al., 2008). The phosphorylated Tyr1175 (pY1175) site at the CTD of VEGFR-2 can bind the Src homology domain-2 (SH2) of the adaptor protein SHB and regulate cell migration caused by VEGF (Holmqvist et al., 2004; Figure 2). The downregulated expression of SHB can prevent VEGF/VEGFR-2-mediated cell scaffold reorganization, migration, and PI3K activation (Masoumi Moghaddam et al., 2012; Park et al., 2018). The phosphorylated Tyr951 (pY951) site at the TKD of VEGFR-2 can bind T-cell-specific adapter (TSAd) in the vascular endothelial cells of tumor tissue (Matsumoto et al., 2005), activate formation of TSAd and Src complex, and subsequently regulate cell migration. Site-directed mutation of Tyr951 on VEGFR-2 can inhibit VEGF-mediated cytoskeletal reorganization and migration (Cebe-Suarez et al., 2006). While the phosphorylated Tyr1214 (pY1214) site at the C-terminus domain of VEGFR-2 is involved in the remodeling of actin by VEGFR-2 mediated NCK/Src-p21/Cdc42-SAPK2/p38-MAPK pathway (Lamalice et al., 2004, 2006).

### VEGFR-2 Enhances Vascular Permeability

The VEGF-A acts as a vascular permeability factor and its signal can activate endothelial nitric oxide synthase (eNOS) to create NO, and then change vascular permeability (Holmes et al., 2007). VEGF-activated VEGFR-2 can stimulate vascular endothelial cells to release NO, in which the phosphorylation of the Try801 (pY801) residue at the JMD of VEGFR-2 is necessary for the release of NO induced by VEGF, and the pY801 activates eNOS mainly by the PKC-PI3K/Akt pathway (Blanes et al., 2007; Figure 2). VEGFR-2 mediated signals can promote eNOS binding to its molecular chaperone heat shock protein 90 (Hsp90), and then enhance the release of NO by endothelial cells (Duval et al., 2007). VEGFR-2 can also mediate effect of lowering blood pressure via increased permeability of blood vessels and relaxed of blood vessels caused by VEGF-A (Gliki et al., 2001).

## VEGFR-2 Physiological Functions and Pathological Roles

In organism, many angiogenic proteins are involved in the stimulation of angiogenesis including EphrinB2/EphB4 (Groppa et al., 2018), fibroblast growth factors (FGFs) (Maddaluno et al., 2017), VEGFs/VEGFR-2 (Basagiannis et al., 2016), angiopoietin/Tie receptors (Zhang et al., 2019), and platelet-derived growth factors (PDGFs/PDGFRs) (Zhang et al., 2009; Manzat Saplacan et al., 2017). Among the angiogenic proteins, VEGFs/VEGFR-2 is a crucial regulator of physiological vasculogenesis and angiogenesis in early embryonic and adult stages and pathological angiogenesis in tumorigenesis.

### Physiological Functions of VEGFR-2

VEGFR-2 mediates the main physiological functions of VEGF. VEGF, the vascular permeability factor (VPF), is an essential and crucial growth factor for vascular endothelial cells, and plays various roles in cardiovascular system (Wang et al., 2019), central nervous system (Bellon et al., 2010; Luck et al., 2019), hematopoiesis (Hooper et al., 2009), development (Karaman et al., 2018), and tumorigenesis (Volz et al., 2020; Zhong et al., 2020). Despite having so many physiological functions, the main physiological functions of VEGF on endothelial cells are almost all achieved by activating VEGFR-2, including stimulating endothelial cell proliferation, increasing vascular permeability, and chemotaxis to endothelial cells, etc. Investigations showed that VEGFR-2 is the main receptor to mediate the increase in vascular permeability by VEGF (Koch and Claesson-Welsh, 2012; Smith et al., 2020). VEGFR-2 is required for VEGF-mediated signaling and regulating. The phosphorylation of VEGFR-2 can be found on the surface of whole cell. Several important phosphorylation sites, Try951, Try1054, Try1059, and Try1175 had been proved to be involved in this process by binding SHB (Holmqvist et al., 2004; Smith et al., 2020), SCK (Warner et al., 2000), PLCγ (Takahashi et al., 2001), PI3K (Blanes et al., 2007), and TASD (Matsumoto et al., 2005) and activating their respective signal pathway.

VEGFR-2 plays a very important role in embryonic development. VEGF-activated VEGFR-2 stimulates endothelial cell proliferation and is crucial to the development of the embryonic vascular system and hematopoietic system (Hooper et al., 2009; Han et al., 2018). During embryonic development, the VEGFR-2 signaling pathway is involved in the proliferation, growth, and migration of hematopoietic and early endothelial cells (angioblasts) (Dias et al., 2000; LeCouter et al., 2004). Several studies showed that the VEGF/VEGFR-2 signaling directly regulates the development and function of neurons, e.g., increased axon branching (Luck et al., 2019).

### Pathological Rroles of VEGF-Activated VEGFR-2

Though the VEGF/VEGFR-2 system plays important functions in normal physiological condition, de-regulation of the VEGF/VEGFR-2 implicates directly in various diseases, and dysfunctional VEGFR-2 can cause developmental disorders of the vascular system and hematopoietic system during embryonic development (Shibuya, 2001; Zeng et al., 2001).

VEGF-activated VEGFR-2 plays important roles in mediating the formation of new blood vessels under various pathological conditions and processes, including wound healing, rheumatoid arthritis (RA), diabetic retinopathy (DR), Alzheimer’s disease (AD), small vessel disease, coronary heart disease (CHD), and cancer owing to its complicated molecular and structural characteristics. The VEGFA/VEGFR-2 signal transduction leads to endothelial cell proliferation, migration, survival and new vessel formation involved in angiogenesis, and has been implicated in pathogenesis of several diseases, e.g., inflammation, cancers, ophthalmic diseases, and neurological diseases (Louveau et al., 2015; Ferrara and Adamis, 2016; Ma et al., 2017).

VEGFR-2 is required for cardiovascular system diseases. Investigations showed that VEGFR-2-deficient mice die in early embryo for an early defect in the development of hematopoietic and endothelial cells (Shalaby et al., 1995; Carmeliet et al., 1996; Ferrara et al., 1996). VEGFR-2 is also involved in tumor angiogenesis and lymphatic development via recruiting endothelial cells (Dumont et al., 1998; Rafii et al., 2002). Studies have reported that the expression of Ets-1 and Flk-1 (mouse VEGFR-2) are highly correlated in angiogenesis and tumor angiogenesis, and are involved in stem cell leukemia (Elvert et al., 2003).

VEGFR-2 is an important target of anti-tumor angiogenesis. VEGF secreted by tumor cells activates its receptor VEGFR-2, and they subsequently promote vascular growth and supply the oxygen and nutrition into the hypoxic areas of tumor tissues (Lugano et al., 2020). VEGF-activated VEGFR-2 mediate the phosphorylation of many proteins in the downstream signaling pathways, e.g., Akt (protein kinase B), mTOR (mammalian target of rapamycin), Erk1/2 (extracellular signal-regulated kinase 1/2), FAK (focal adhesion kinase), and p70S6K (ribosomal protein S6 kinase), and promotes tumor angiogenesis (Karar and Maity, 2011; Lechertier and Hodivala-Dilke, 2012; Ji et al., 2018). Therefore, VEGFR-2 functions as an important target for anti-tumor therapy (Weis and Cheresh, 2011; Fallah et al., 2019).

## Conclusion and Perspectives

Vascular endothelial growth factors, as the key regulators, are involved in vasculogenesis, angiogenesis, and hematopoiesis during development. Thay play their roles by binding to and activating their tyrosine kinase receptors (VEGFRs) on the cell surface. In VEGF/VEGFR-2 system, the VEGF binding to the Ig-like subdomains 2 and 3 (IgD2 and IgD3) of VEGFR-2 induces the dimerization of the receptor, the activation and trans-autophosphorylation of the tyrosine kinase, and then the initiation of the intracellular signaling cascades, finally is involved in proliferation, survival, migration, and permeability of vascular endothelial cells.

The interaction of VEGF and VEGFR-2 is crucial for the dimerization of VEGFR-2. In endothelial cell, the signaling by VEGFR-2 requires VEGF-mediated dimerization with precise positioning of VEGFR-2 subunits in active dimers. In VEGF-A, there are a canonical cysteine knot that consists of three intramolecular disulfide bridges Cys263-Cys308, Cys267-Cys310, and Cys232-Cys274, and two receptor binding sites that bind to each VEGFR-2 of the dimer and dimerization interface sites, which are critical for the ligand binding to VEGFR-2. In VGEFR2 Ig-like subdomains, the IgD2 and IgD3 are involved in the VEGF binding to its receptor. The dimeric VEGF/VEGFR-2 complexes subsequently induce the dimerization of the receptor.

The dimerization of VEGFAR-2 is required for activation of VEGFR-2. The dimeric VEGF ligands binding to diffusing monomeric VEGFR-2 promotes dimerization of the later, and then stimulates the activation of VEGFR-2. It is reported that ligand-induced VEGFR-2 phosphorylation is increased as much as 10-fold compared to the phosphorylation in the absence of ligand (Sarabipour et al., 2016). In the active homotypic VEGFR-2-VEGFR-2, the IgD4∼7 were proved to be critical for efficient phosphorylation of the VEGFR-2 in the presence of VEGF, and the TMD dimer upon ligand binding increases VEGFR-2 phosphorylation and stabilizes the VEGFR-2 dimers, indicating that VEGF binding and VEGFR-2 dimerization are required for its activation.

The VEGFR-2-mediated intracellular signaling are involved in cell survival, proliferation, migration, and vascular permeability. VEGFR-2 is the primary mediator of the physiological effects of VEGF in angiogenesis. The VEGF binding to VEGFR-2 induces autophosphorylation of specific tyrosine residues in the cytoplasmic domain of VEGFR-2, including the Try801 on JMD involved in cell permeability and proliferation by mediating the PLCγ-PKC, then eNOS-NO or MEK-ERK, respectively, the Try951 on KID mediating the cell survival and permeability via the TSAd-Src-PI3K-Akt pathway, the Try1054 and Try1059 on TKD2 increasing VEGFR-2 kinase activity, the Try1175 involved in cell permeability, proliferation, and migration by regulating PLCγ-PKC, SHB-FAK-paxillin, and SHB-PI3K-Rac pathways, and the Try1214 mediates cell migration through NCK-p38-MAPKAPK2/3 pathway. These signaling networks mediated by VEGF/VEGFR-2 are involved in regulating the process of angiogenesis, and controlling endothelial cell survival, proliferation and motility, and vascular fenestration and permeabilization.

The VEGFR-2-targeted therapy strategies will contribution to clinical treatment of disease. So far, the in-depth study of the structure and signal transduction of VEGFR-2 has made the mechanism of related pathogenesis further elucidated and effectively treated. VEGFR-2-targeted therapy strategies have been widely explored and applied clinically in cancer treatment. VEGF/VEGFR-2 signal transduction pathway is used in anti-tumor angiogenesis. VEGF/VEGFR-2 system is involved in various pathological conditions and processes, especially in tumorigenesis, owing to its complicated molecular structure and signal transduction, indicating these diseases can be considered to employ the strategy of the VEGFR-2-targeted therapy. The tumor microenvironment stimulates the specific expression of VEGF and VEGFR-2 in tumor cells and endothelial cells around the tumor, making the expression of VEGF and VEGFR-2 significantly higher than in normal tissues, indicating that inhibiting tumor angiogenesis by blocking the VEGF/VEGFR-2 signaling pathway may be an effective anti-cancer treatment strategy, including (a) inhibiting VEGF and VEGFR-2 gene expression level by antisense oligonucleotides (ASOs), RNA interference (RNAi), and ribozyme (Rz) (Marchand et al., 2002); (b) blocking the binding of VEGF and VEGFR-2 in protein level through neutralizing antibodies (nAbs), soluble VEGFR-2 (sVEGFR-2), and small molecule inhibitor VEGF/VEGFR-2 tyrosine kinase signaling pathway (Jain et al., 2006); and (c) destroying vascular endothelial cells through directed therapy by VEGF combined with small molecule toxic substances or VEGFR-2 monoclonal antibody (mAb) cross-linked with drugs and killing or inhibiting the growth of vascular endothelial cells expressing VEGFR-2 (Stopeck et al., 2002).

## Author Contributions

XW, AMB, GS, and BM contributed to manuscript writing and language improving. BM and XW designed and made the figures and wrote and edited the review. All authors contributed to the article and approved the submitted version.

## Conflict of Interest

The authors declare that the research was conducted in the absence of any commercial or financial relationships that could be construed as a potential conflict of interest.
